# Growth and capacity for cost‐effectiveness analysis in Africa

**DOI:** 10.1002/hec.4029

**Published:** 2020-05-15

**Authors:** Ari D. Panzer, Joanna G. Emerson, Brittany D'Cruz, Avnee Patel, Saudamini Dabak, Wanrudee Isaranuwatchai, Yot Teerawattananon, Daniel A. Ollendorf, Peter J. Neumann, David D. Kim

**Affiliations:** ^1^ Center for the Evaluation of Value and Risk in Health Tufts Medical Center Boston Massachusetts USA; ^2^ Health Intervention and Technology Assessment Program (HITAP) Ministry of Public Health Nonthaburi Thailand; ^3^ Institute of Health Policy, Management and Evaluation University of Toronto Toronto Ontario Canada; ^4^ The Saw Swee Hock School of Public Health National University of Singapore Singapore

**Keywords:** Africa, cost‐effectiveness analysis, economic evaluation, network analysis, universal health coverage

## Abstract

As economic evaluation becomes increasingly essential to support universal health coverage (UHC), we aim to understand the growth, characteristics, and quality of cost‐effectiveness analyses (CEA) conducted for Africa and to assess institutional capacity and relationship patterns among authors. We searched the Tufts Medical Center CEA Registries and four databases to identify CEAs for Africa. After extracting relevant information, we examined study characteristics, cost‐effectiveness ratios, individual and institutional contribution to the literature, and network dyads at the author, institution, and country levels. The 358 identified CEAs for Africa primarily focused on sub‐Saharan Africa (96%) and interventions for communicable diseases (77%). Of 2,121 intervention‐specific ratios, 8% were deemed cost‐saving, and most evaluated immunizations strategies. As 64% of studies included at least one African author, we observed widespread collaboration among international researchers and institutions. However, only 23% of first authors were affiliated with African institutions. The top producers of CEAs among African institutions are more adherent to methodological and reporting guidelines. Although economic evidence in Africa has grown substantially, the capacity for generating such evidence remains limited. Increasing the ability of regional institutions to produce high‐quality evidence and facilitate knowledge transfer among African institutions has the potential to inform prioritization decisions for designing UHC.

## 1. INTRODUCTION

Health technology assessment (HTA) could help to identify and prioritize cost‐effective interventions to support universal health coverage (UHC; World Health Organization, 2014a; World Health Organization, 2014b). However, conducting economic evaluations, such as cost‐effectiveness analysis (CEA), can be challenging because these analyses require additional training, appropriate data inputs, and contextual considerations (Drummond, Sculpher, Claxton, Stoddart, & Torrance, 2015; Sanders et al., 2016). As such, HTA agencies have not yet been formalized in most low‐ and middle‐income countries (LMIC), particularly in Africa, where nearly half of countries in the sub‐Saharan region are classified as low‐income, making prioritization of constrained resources critical (Bank, 2019; Chalkidou et al., 2016; Li, Hernandez‐Villafuerte, Towse, Vlad, & Chalkidou, 2016). Also, there are uncertainties around whether these countries have the technical capacity and organizational support to generate economic evidence suitable to their contexts and settings (Babigumira Jenny, Bartlein, Stergachis, & Garrison, 2016; Chuang, Chuang, Ho, & Ho, 2011; Doherty, Wilkinson, Edoka, & Hofman, 2017; Hofman, Kanyengo, Rapp, & Kotzin, 2009; Pitt, Goodman, & Hanson, 2016; Tantivess, Chalkidou, Tritasavit, & Teerawattananon, 2017; Wagstaff & Culyer, 2011).

With the substantial growth and the increased accessibility of such information, (Center For The Evaluation Of Value And Risk In Health (CEVR), Tufts Medical Center (2019a,b) a comprehensive review of economic evaluations conducted for Africa would shed light on the growth, characteristics, and quality of cost‐effectiveness studies as well as identify gaps in the field. Previous bibliometric analyses of economic evaluations in Africa are limited in scope and outdated, highlighting the need for comprehensive and updated analyses (Chuang et al., 2011; Hernandez‐Villafuerte, Li, & Hofman, 2016; Hofman et al., 2009; Odame, 2013). Additionally, given the extensive benefits of the increasing level of international collaborations to generate and use economic evidence in global health policy (Atkinson, Batchelor, & Parsons, 1998; Ettarh, 2015; Fershtman & Gandal, 2011; Hoekman, Frenken, & van Oort, 2008; Imperial College London & Center for Global Development, 2019; Lachat et al., 2015), a formal assessment of institutional capacity and relationship patterns among authors can identify potential opportunities for capacity building and future collaboration.

## 2. METHODS

### 2.1. Literature search

To identify CEAs conducted for African countries (Appendix S1), we analyzed the Tufts Medical Center's CEA Registry (a database of cost‐per‐quality‐adjusted‐life‐year [QALY] studies) and Global Health CEA Registry (a database of cost‐per‐disability‐adjusted‐life‐year [DALY] studies; Center For The Evaluation Of Value And Risk In Health (CEVR), Tufts Medical Center, 2019a,b). Both registries contain information from English‐language, original CEAs indexed in PubMed, and report at least one cost‐per‐QALY gained or cost‐per‐DALY averted ratio. Previous work documents additional methodology in detail (Butt, Liu, Kim, & Neumann, 2019; Neumann et al., 2016). To supplement the registries, we also searched four other databases (i.e., the National Health Service Economic Evaluation Database, Nixon et al., 2000; Embase, Elsevier, 2018; EconLit, American Economic Association, 2019; and Web of Science, Clarivate Analytics, 2019) with a defined set of search terms available in Appendix S2.

### 2.2. Screening and data extraction

Two researchers (A. D. P. and J. G. E.) independently screened the title and abstract of each article identified from non‐Tufts registry sources and determined whether the study met our inclusion criteria. Appendix S3 provides the full inclusion criteria and a PRISMA diagram detailing the screening process and results. We then conducted a full‐text review of the final sample using a standardized data collection form to extract variables relating to the study and author characteristics. Appendix S4 provides a full list of extracted variables and descriptions.

### 2.3. Data analysis

We examined the association between the number of available studies and study characteristics, including outcome measure, disease area, and intervention type at three time periods (pre‐2010, 2010–2014, and 2015–2017). We also analyzed whether the reported cost‐effectiveness ratios varied across the intervention types.

To determine the top contributing individuals and institutions, we ranked authors and institutions by the number of total published studies and examined the proportion of first authors with an African affiliation. We compared study characteristics published by the top three (contributing to more than 10 studies) versus other African institutions to evaluate whether the increased capacity and experience is associated with improved study quality. As a sensitivity analysis, we examined a study's adherence to the iDSI methodological and reporting guidelines (Emerson et al., 2019; Wilkinson et al., 2016). We performed chi‐square test or independent samples *t‐* test to evaluate a statistically significant difference.

We generated author dyads for each study and counted the frequency of each dyad to identify the pairs of authors most frequently collaborating with one another. Similarly, we repeated this approach to create institution and country dyads and quantified the extent of cross‐national and within‐Africa collaboration. We provide a worked example for more thorough explanation of dyad generation in Appendix S5.

All analyses were conducted in Stata 15 (StataCorp, 2017) and RStudio (RStudio, 2018).

## 3. RESULTS

### 3.1. Study sample

We initially identified 1,251 studies indexed across the six databases and then removed 802 studies, including duplicates (*n* = 421) and those not meeting the inclusion criteria (*n* = 381). We excluded an additional 91 studies, which did not meet the inclusion criteria after reviewing the full text, resulting in 358 studies in the final sample, with 94% (*n* = 339) coming from the CEA and Global Health CEA registries.

### 3.2. Study characteristics of CEAs conducted for Africa

**FIGURE 1:**
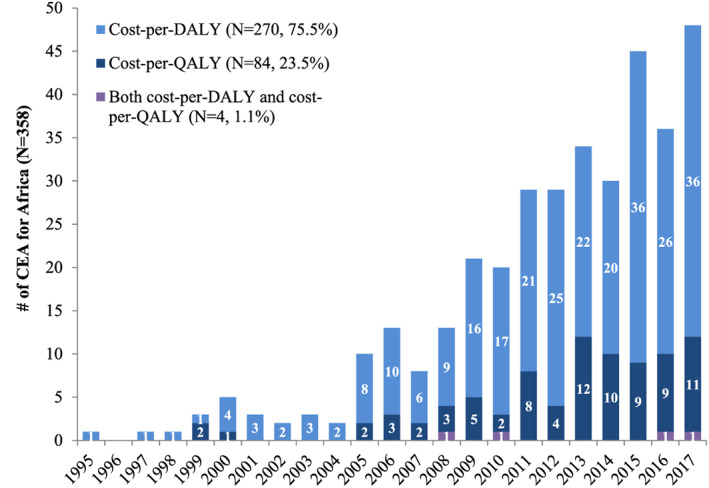
Growth of African cost‐effectiveness analyses over time. *Note*: This figure shows the growth of African CEAs over time. The first CEA in our sample was published in 1992, which is not shown in the Figure. Studies reporting both QALYs and DALYs as health outcome measures are categorized separately. [Colour figure can be viewed at wileyonlinelibrary.com]

**FIGURE 2:**
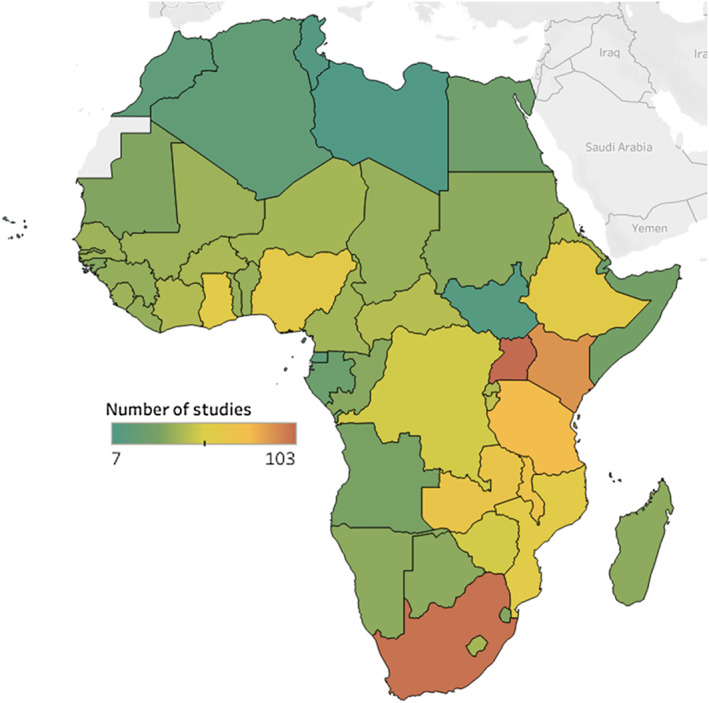
Number of cost‐effectiveness analyses by African countries. *Note*: Cost‐effectiveness analyses include cost‐per‐QALY and cost‐per‐DALY studies. (See Appendix S6: Figures B and C for the literature‐specific maps.) In this figure, green indicates a relatively low number of studies while red indicates a relatively high number of studies. For example, the top three countries include Uganda (*N* = 103), South Africa (*N* = 101), and Kenya (*N* = 91), whereas the lowest three are Libya (*N* = 6), Tunisia (*N* = 9), and South Sudan (*N* = 10) [Colour figure can be viewed at wileyonlinelibrary.com]

The number of African CEAs published per year continuously increased over time (Figure 1). Among the 358 included studies, most (*n* = 344, 96%) focused on countries in sub‐Saharan Africa, whereas relatively few (*n* = 56, 16%) focused on countries in North Africa (Table 1). The DALY was a more prevalent health outcome measure (*n* = 274, 77%) than the QALY (*n* = 88, 25%). Studies most frequently assessed pharmaceutical interventions (*n* = 152, 42%) or immunizations (*n* = 78, 22%). Communicable diseases remained the primary focus of studies, including those published in the most recent period between 2015 and 2017. Uganda (*n* = 103), South Africa (*n* = 101), and Kenya (*n* = 91) were the three most studied countries (Figure 2; Appendix S7: Table A). Among 2,121 intervention‐specificcost‐effectiveness ratios extracted from 358 studies, 8% (*n* = 162) were deemed cost‐saving, and most of these evaluated immunizations, pharmaceuticals, or screening strategies. A greater proportion of cost‐per‐QALY ratios were reported to be cost‐saving compared with cost‐per‐DALY ratios (*n* = 34, 14% vs. *n* = 128, 7%; Appendix S7: Table B).

**TABLE 1:** Characteristics of economic evaluations in Africa

	Pre‐2010^b^		2010–2014^b^		2015‐2017^b^		Overall^b^	
	Number of studies	%	Number of studies	%	Number of studies	%	Number of studies	%
All studies	87	24%	142	40%	129	36%	358	100%
Health outcome measure^a^								
Cost/DALY	68	78%	106	75%	100	78%	274	77%
Cost/QALY	20	23%	37	26%	31	24%	88	25%
GBD super region^a^								
Sub‐Saharan Africa	86	99%	136	96%	122	95%	344	96%
North Africa	15	17%	23	16%	18	14%	56	16%
Prevention level^a^								
Primary	46	53%	68	48%	58	45%	172	48%
Secondary	20	23%	23	16%	21	16%	64	18%
Tertiary	36	41%	62	44%	59	46%	157	44%
Country vs. regional ratios^a^								
Country‐specific	70	80%	120	85%	113	88%	303	85%
Regional	18	21%	28	20%	16	12%	62	17%
Study Perspective								
Societal/limited societal	22	25%	27	19%	27	21%	76	21%
Health care payer/sector	64	74%	108	76%	99	77%	271	76%
Other/could not be determined	1	1%	7	5%	3	2%	11	3%
Top GBD disease categories^a^								
HIV/AIDS and tuberculosis	33	38%	51	36%	41	32%	125	35%
Diarrhea, lower respiratory infections, meningitis, and other common infectious diseases	10	11%	26	18%	20	16%	56	16%
Neglected tropical diseases and malaria	15	17%	17	12%	15	12%	47	13%
Other communicable, maternal, neonatal, and nutritional disorders	13	15%	10	7%	10	8%	33	9%
Cardiovascular and circulatory diseases	3	3%	8	6%	6	5%	17	5%
Maternal disorders	2	2%	3	2%	10	8%	15	4%
Nutritional deficiencies	4	5%	5	4%	3	2%	12	3%
Other noncommunicable diseases	2	2%	4	3%	5	4%	11	3%
Neoplasms	3	3%	5	4%	2	2%	10	3%
Diabetes, urogenital, blood, and endocrine diseases	0	0%	3	2%	6	5%	9	3%
Other diseases	10	11%	22	15%	18	14%	50	14%
Top interventions^a^								
Pharmaceutical	43	49%	61	43%	48	37%	152	42%
Immunization	19	22%	31	22%	28	22%	78	22%
Care delivery	17	20%	19	13%	26	20%	62	17%
Health education or behavior	13	15%	15	11%	19	15%	47	13%
Screening	12	14%	19	13%	15	12%	46	13%
Maternal and neonatal	9	10%	13	9%	11	9%	33	9%
Diagnostic	4	5%	12	8%	10	8%	26	7%
Surgical	4	5%	14	10%	8	6%	26	7%
Other interventions	23	26%	31	22%	28	22%	82	23%
Study sponsor^a^								
Government/academic	53	61%	70	49%	54	42%	177	49%
Foundation	25	29%	43	30%	41	32%	109	30%
Pharma/device company	6	7%	8	6%	10	8%	24	7%
Could not be determined/none	18	21%	38	27%	27	21%	83	23%
Other	12	14%	20	14%	18	14%	50	14%

a Not mutually exclusive categories.

b Proportions are based on the total number of studies during each time point.

### 3.3. Institutional capacity and collaboration patterns

African investigators and institutions have maintained a strong presence in the production of CEAs targeted to African countries, with 64% of studies including at least one author with an African affiliation and 51% including more than one. However, less than one‐quarter of *first* authors (23%) reported an African affiliation. At the institution level, the London School of Hygiene & Tropical Medicine, UK, contributed the most studies (*n* = 58), whereas Makerere University, Uganda (*n* = 25), University of Cape Town, South Africa (*n* = 23), and University of the Witwatersrand, South Africa (*n* = 20), were the top three African institutions (Table 2).

**TABLE 2:** Institutional capacity

	Number of studies	% of total studies (*n* = 358)
Any author		
African affiliation	228	64%
No African affiliation	130	36%
Multiple African affiliations	183	51%
First author		
African affiliation	81	23%
No African affiliation	277	77%
Senior author		
African affiliation	92	26%
No African affiliation	266	74%
Top 5 worldwide institutions		
London School of Hygiene & Tropical Medicine	58	16%
World Health Organization	42	12%
Harvard University	35	10%
Johns Hopkins University	34	9%
U.S. Centers for Disease Control and Prevention	28	8%
Top 5 African institutions		
Makerere University	25	7%
University of Cape Town	23	7%
University of the Witwatersrand	20	6%
Kenya Medical Research Institute	7	2%
Tanzania Ministry of Health	7	2%
Top 5 worldwide authors^a^		
Kahn, James G. (University of California, San Francisco)	15	4%
Marseille, Elliot (University of California, San Francisco)	10	3%
Baltussen, Rob (Radboud University)	9	3%
Chisholm, Dan (World Health Organization)	9	3%
Hallett, Timothy B. (Imperial College London)	8	2%
Top 5 African‐based authors^a^ ^,^ ^b^		
Lamorde, Mohammed (Makerere University)	8	2%
Kuznik, Andreas (Bayero University; Makerere University; Regeneron)	7	2%
Manabe, Yukari C. (Johns Hopkins University)	7	2%
Cleary, Susan M. (University of Cape Town)	6	2%
Mermin, Jonathan (U.S. Centers for Disease Control and Prevention)	6	2%
Top 5 African countries		
South Africa	53	15%
Uganda	47	13%
Kenya	32	9%
Nigeria	23	6%
Tanzania	23	6%

a Rankings based on co‐authorship irrespective of author order.

b We relied on the information available in each study to determine each author's institution of affiliation and country of affiliation. We considered authors to have an “African‐based affiliation” if the country of affiliation reported for their affiliated institution was a country in Africa. For example, since the U.S. Centers for Disease Control and Prevention have programs and placements in Kenya and Uganda, we consider these to be “African‐based affiliations.”

Although researchers from 76 different countries contributed to generating economic evidence for Africa, research collaborations tended to be based in the same country. When African researchers collaborated internationally, they most frequently worked with researchers based in the United States, followed by the United Kingdom (Appendix S6: Figure A). Within the African region, authors based in Uganda and Nigeria worked together most frequently (*n* = 6), highlighting the presence of regional collaboration within Africa. There was little collaboration between researchers in countries in North Africa and sub‐Saharan Africa (Appendix S6: Figure B, Appendix S7: Table C).

### 3.4. Study characteristics by top African institutions

The characteristics of CEAs produced by top institutions (ranked by number of published CEAs) varied in a number of ways, compared with those by other African institutions (Appendix S7: Table G). The top three institutions more frequently reported QALYs as a health outcome measure (35% vs. 15%, *p* < .001) and focused most frequently on HIV/AIDs and tuberculosis (53% vs. 31%, *p* < .001). They were also more likely to perform advanced analyses, such as probabilistic sensitivity analysis (60% vs. 42%, *p* < .05), and published CEAs with a higher average quality score, though the difference was not statistically significant (mean quality score: 5.0 vs. 4.8, *p* = .194). Top institutions were also more adherent to the iDSI reference case's methodological (65% vs. 57%, *p* < .001) and reporting guidelines (76% vs. 72%, *p* < .05) and surpassed the average adherence scores across all cost‐per‐DALY studies evaluated relative to the iDSI reference case (65% vs. 60% for methodological standard; 76% vs. 74%for reporting standard; Table 3; Emerson et al., 2019).

**TABLE 3:** Adherence to the iDSI reference case among CEAs produced by top and other African institutions

iDSI reference case adherence score principle^b^	Top African institutions^a^ (number of CEAs: 31)	Other African institutions (number of CEAs: 90)	Difference (95% CI)	*p* value	Full adherence score sample from Emerson et al., 2019 (number of CEAs: 398)
	Mean (SE)	Mean (SE)			Mean
Reporting					
Overall	76.34 (1.22)	72.38 (.93)	3.96 (.51, 7.41)	.0247	73.9
Budget impact	9.68 (5.40)	4.44 (2.18)	5.23 (−4.43, 14.89)	.286	9.3
Comparator	98.39 (1.61)	94.44 (1.67)	3.94 (−1.99, 9.88)	.191	96.86
Costs	51.61 (1.61)	52.22 (1.35)	−0.61 (−5.53, 4.32)	.807	53.52
Equity	9.68 (5.40)	3.33 (1.90)	6.34 (−2.61, 15.30)	.163	6.53
Evidence	96.77 (3.23)	96.67 (1.90)	0.11 (−7.33, 7.54)	.977	95.23
Heterogeneity	38.71 (8.89)	43.33 (5.25)	−4.62 (−25.14, 15.89)	.656	37.19
Outcome	45.16 (9.09)	50 (5.30)	−4.84 (−25.60, 15.93)	.645	53.52
Perspective	96.77 (3.23)	81.11 (4.15)	15.66 (1.14, 30.18)	.0347	85.43
Time horizon	90.32 (3.61)	78.89 (3.53)	11.43 (−1.22, 24.08)	.076	81.91
Transparency	89.86 (1.90)	84.76 (1.62)	5.10 (−.82, 11.02)	.09	86.32
Uncertainty	100	100	‐	‐	100
Methods					
Overall	65.37 (1.87)	56.73 (1.27)	8.64 (3.84, 13.44)	.0005	59.63
Budget impact	6.45 (4.49)	5.56 (2.43)	0.90 (−8.81, 10.6)	.855	10.05
Comparator	51.61 (9.12)	32.22 (4.95)	19.39 (−.40, 39.18)	.055	35.68
Costs	72.58 (5.60)	70.56 (3.52)	2.03 (−11.53, 15.58)	.768	65.33
Equity	9.68 (5.40)	3.33 (1.90)	6.34 (−2.61, 15.30)	.163	6.53
Evidence	74.19 (7.99)	62.22 (5.14)	11.97 (−7.70,31.65)	.231	43.12
Heterogeneity	38.71 (8.89)	43.33 (5.25)	−4.62 (−25.4, 15.89)	.656	37.19
Outcome	100	100	‐	‐	100
Perspective	61.29 (4.47)	58.89 (2.57)	2.40 (−7.70, 12.50)	.639	63.82
Time horizon	69.89 (4.97)	47.78 (3.61)	22.11 (8.61, 35.62)	.0015	57.2
Transparency	95.70 (2.04)	86.67 (1.96)	9.03 (2.01, 16.06)	.012	89.45
Uncertainty	65.30 (4.50)	56.30 (2.82)	9.30 (−1.56, 20.15)	.093	57.04

a We consider CEAs co‐authored by the top three African institutions (those contributing to more than 10 studies) “Top African Institution Studies,” whereas all other studies co‐authored by other African institutions are considered “Other African Institution Studies.”

b This analysis includes only studies that included methods and reporting reference case adherence scores generated by Emerson et al. (2019). Scores are based on “required” methodological specifications (*n* = 19) and reporting standards (*n* = 21). Raw scores are converted to a percentage of total possible points, measured as normalized adherence score (0% = no adherence, i.e., no requirements adhered to; 100% = full adherence, all requirements adhered to). We provide the methodological summary in Appendix S4.

## 4. DISCUSSION

Economic evidence for assessing the value of health interventions in Africa has grown substantially over the past decade and is available for every African country across various disease areas. Though it can be challenging to understand the value offered by potentially outdated studies, the vast majority included have been published since 2008 (85%).This progress highlights the potential to inform prioritization decisions for designing UHC packages. Our study, however, finds that the volume of available evidence varies markedly by country.

Compared with economic evaluations conducted for other regions (Butt et al., 2019; Neumann et al., 2018; Pitt et al., 2016), African studies remain more focused on interventions for communicable diseases (77% of published CEAs); Global Burden of Disease Collaborative Network, 2018). The focus of funders and international organizations might also contribute to a disproportionately large number of economic evaluations in communicable diseases, relative to the disease burden (GAVI: The Vaccine Alliance, 2019). With the growing burden of non‐communicable diseases (NCD) (Global Burden of Disease Collaborative Network, 2018), more attention from ministries of health, funders, and international organizations is needed to generate economic evidence for interventions targeting NCDs.

The capacity for generating such evidence within most African institutions remains somewhat limited, highlighting the need to invest in capacity‐building activities while continuing to leverage existing international collaborations. A good model of such collaboration is the long‐standing partnership between the Kenya Medical Research Institute and the Wellcome Trust to improve health across the region and develop African scientific leaders (2019).

The top African institutions have produced economic evidence with better methodological quality and advanced analysis. Building capacity around these institutions (e.g., the development of regional hubs) would be valuable to generate more transferable economic evidence across African countries, considering epidemiologic and economic similarities in the region. Also, in the absence of local guidelines, funders may request economic evaluations to follow the practice guidelines when commissioning economic evaluations to ensure quality and comparability of studies.

Our findings show the widespread collaboration among international researchers, yet collaboration patterns are not equivalent across the African regions. This pattern perhaps reflects broader systemic differences between the regions, such as donor funding, political environments, and existing partnerships between academics and the local government. Encouraging knowledge transfer among African institutions—through establishing HTA network in Africa, such as HTAsiaLink (2019), and using the African Health Economics and Policy Association (AfHEA; 2019) as a platform—could be an essential next step to develop technical collaboration.

Our study has some limitations. First, we only included published cost‐per‐QALY or cost‐per‐DALY studies, excluding other types of economic analyses (e.g., benefit–cost analyses or cost‐per‐natural‐unit studies). A previous study suggested that cost‐utility analyses accounted for at least half of economic evaluations, whereas the majority of other studies are other types of CEAs (Pitt et al., 2016). In the context of resource prioritization, however, studies reporting outcomes in QALYs or DALYs are essential so that interventions for different disease areas can be comparable and informative for designing UHC. Also, we did not capture the non‐English language and non‐published economic analyses, such as government documents or the gray literature. Considering that all economic evaluations addressing LMICs were published in English (Pitt et al., 2016) and that only one HTA agency has been formalized in Africa (i.e., Tunisia; Egypt also incorporates some elements of HTA) (INEAS, 2020; Elsisi et al., 2013; El‐Sisi & Eldessouki, 2018), the number of potentially excluded studies is likely to be minimal. Finally, despite its use in the past (Butt et al., 2019; Neumann et al., 2016; Neumann, Fang, & Cohen, 2009; Neumann, Stone, Chapman, Sandberg, & Bell, 2000), the quality rating used in this study is a subjective measure that may not fully capture the appropriateness of input estimates. An alternative approach is to measure each article's adherence to reporting and methodological guidelines (Emerson et al., 2019).

## 5. CONCLUSION

While there has been a rapid growth of economic evidence for African countries, this review highlights a need for more information to help inform UHC development efforts and address changing disease burden patterns. Increasing the capacity of regional institutions to produce high‐quality economic evidence and facilitating knowledge transfer among African institutes has the potential to generate more transferable economic evidence and promote better decision‐making by providing policymakers with more high‐quality, contextually relevant information.

## AUTHORS' CONTRIBUTIONS

Y. T., P. J. N., and D. D. K. conceived and designed the study. All authors discussed, critically revised, and approved the study protocol. D. D. K. was responsible for the organization and conduct of the study and supervised it. A. D. P. was responsible for the data collection and analysis. J. G. E. and B. D. contributed to the data collection and analysis. A. D. P. and D. D. K. drafted the first version of the manuscript. All authors elaborated, discussed, and approved the final version of the manuscript for publication.

## CONFLICT OF INTEREST

The authors have declared that no competing interests exist.[Correction added on 11 June 2020 after first online publication. The statement in the last paragraph of the Discussion section has been corrected and a reference citation has been added as well in this current version.]

## Supporting information

Data S1 Supporting Information
