# Supplementary material for: Growth and capacity for cost‐effectiveness analysis in Africa
Source: Health Econ. 2020 May 15;29(8):945–54. doi: 10.1002/hec.4029 (PMC7383734; doi:10.1002/hec.4029)
Supplement: Supplementary file 1 — Data S1 Supporting Information [file HEC-29-945-s001.docx]

**Supplementary Appendices – Growth and capacity for cost-effectiveness analysis in Africa**

**Appendix S1– List of countries in Africa by region (based on 2016 GBD study)**

North Africa and Middle East

Algeria

Egypt

Libya

Morocco

Sudan

Tunisia

Sub-Saharan Africa

Angola

Benin

Botswana

Burkina Faso

Burundi

Cabo Verde

Cameroon

Central African Republic

Chad

Comoros

Congo Republic

Côte d’Ivoire

Democratic Republic of the Congo

Djibouti

Equatorial Guinea

Eritrea

Ethiopia

Gabon

Ghana

Guinea

Guinea-Bissau

Kenya

Lesotho

Liberia

Madagascar

Malawi

Mali

Mauritania

Mozambique

Namibia

Niger

Nigeria

Rwanda

São Tomé and Príncipe

Senegal

Sierra Leone

Somalia

South Africa

South Sudan

Swaziland

Tanzania

The Gambia

Togo

Uganda

Zambia

Zimbabwe

**Appendix S2 – Database search terms**

Since the Global Health CEA Registry already included studies identified from Web of Science, Embase, and EconLit that meet the registry’s inclusion criteria, these additional databases (e.g., Web of Science, Embase, and Econlit) were searched for cost-per-QALY studies to supplement the CEA Registry’s contents. The NHS EED was searched for both cost-per-QALY and cost-per-DALY studies.

Web of Science

Date of Search: 10/5/2018

Search Terms:

(TS=(("qualit* adjust*" NEAR/3 life))) OR (TS=(QALY*)) AND (TS=(Africa) OR TS=(Algeria) OR TS=(Egypt) OR TS=(Libya) OR TS=(Morocco) OR TS=(Sudan) OR TS=(Tunisia) OR TS=(Botswana) OR TS=(Lesotho) OR TS=(Namibia) OR TS=("South Africa") OR TS=(Swaziland) OR TS=(Zimbabwe) OR TS=(Benin) OR TS=("Burkina Faso") OR TS=(Cameroon) OR TS=("Cape Verde") OR TS=("Cabo Verde") OR TS=(Chad) OR TS=("Cote d'Ivoire") OR TS=(Ivory Coast) OR TS=(Gambia) OR TS=(Ghana) OR TS=(Guinea) OR TS=(Guinea-Bissau) OR TS=(Liberia) OR TS=(Mali) OR TS=(Mauritania) OR TS=(Niger) OR TS=(Nigeria) OR TS=("Sao Tome and Principe") OR TS=(Senegal) OR TS=(Sierra Leone) OR TS=(Togo) OR TS=(Burundi) OR TS=(Comoros) OR TS=(Djibouti) OR TS=(Eritrea) OR TS=(Ethiopia) OR TS=(Kenya) OR TS=(Madagascar) OR TS=(Malawi) OR TS=(Mozambique) OR TS=(Rwanda) OR TS=(Somalia) OR TS=("South Sudan") OR TS=(Tanzania) OR TS=(Uganda) OR TS=(Zambia) OR TS=(Angola) OR TS=("Central African Republic") OR TS=(Congo) OR TS=("DR Congo") OR TS=("Congo Republic") OR TS=("Democratic Republic of the Congo") OR TS=("Equatorial Guinea") OR TS=(Gabon))

Limits:

- Databases: Web of Science Core Collection only
- English Language
- Timespan: 1960-2017

EMBASE

Date of Search: 10/5/2018

Search Terms:

(‘quality-adjusted life year’/de) OR (‘qualit*’:ti;ab) OR (‘qaly*’;ti;ab) AND ((Africa:ti,ab) OR (Algeria:ti,ab) OR (Egypt:ti,ab) OR (Libya:ti,ab) OR (Morocco:ti,ab) OR (Sudan:ti,ab) OR (Tunisia:ti,ab) OR (Botswana:ti,ab) OR (Lesotho:ti,ab) OR (Namibia:ti,ab) OR (‘South Africa’:ti,ab) OR (Swaziland:ti,ab) OR (Zimbabwe:ti,ab) OR (Benin:ti,ab) OR ("Burkina Faso":ti,ab) OR (Cameroon:ti,ab) OR (‘Cape Verde’:ti,ab) OR (‘Cabo Verde’:ti,ab) OR (‘Chad’:ti,ab) OR (‘Cote d'Ivoire’:ti,ab) OR (‘Ivory Coast’:ti,ab) OR (Gambia:ti,ab) OR (Ghana:ti,ab) OR (Guinea:ti,ab) OR (Guinea-Bissau:ti,ab) OR (Liberia:ti,ab) OR (Mali:ti,ab) OR (Mauritania:ti,ab) OR (Niger:ti,ab) OR (Nigeria:ti,ab) OR (‘Sao Tome and Principe’:ti,ab) OR (Senegal:ti,ab) OR (‘Sierra Leone’:ti,ab) OR (Togo:ti,ab) OR (Burundi:ti,ab) OR (Comoros:ti,ab) OR (Djibouti:ti,ab) OR (Eritrea:ti,ab) OR (Ethiopia:ti,ab) OR (Kenya:ti,ab) OR (Madagascar:ti,ab) OR (Malawi:ti,ab) OR (Mozambique:ti,ab) OR (Rwanda:ti,ab) OR (Somalia:ti,ab) OR (‘South Sudan’:ti,ab) OR (Tanzania:ti,ab) OR (Uganda:ti,ab) OR (Zambia:ti,ab) OR (Angola:ti,ab) OR (‘Central African Republic’:ti,ab) OR (Congo:ti,ab) OR (‘DR Congo’:ti,ab) OR (‘Congo Republic’:ti,ab) OR (‘Democratic Republic of the Congo’:ti,ab) OR (‘Equatorial Guinea’:ti,ab) OR (Gabon:ti,ab))

Limits:

• Publication Type: Article, Article in Press, Conference paper

• Language: English

ECONLIT

Date of Search: 10/10/2018

Search Terms:

(((TI,AB("qualit* adjust*" NEAR/3 life) OR TI,AB("QALY*")) AND stype.exact("Scholarly Journals" OR "Working Papers")) AND LA(English)) AND (TI,AB(Africa) OR TI,AB(Algeria) OR TI,AB(Egypt) OR TI,AB(Libya) OR TI,AB(Morocco) OR TI,AB(Sudan) OR TI,AB(Tunisia) OR TI,AB(Botswana) OR TI,AB(Lesotho) OR TI,AB(Namibia) OR TI,AB(‘South Africa’) OR TI,AB(Swaziland) OR TI,AB(Zimbabwe) OR TI,AB(Benin) OR TI,AB("Burkina Faso") OR TI,AB(Cameroon) OR TI,AB(‘Cape Verde’) OR TI,AB(‘Cabo Verde’) OR TI,AB(‘Chad’) OR TI,AB(‘Cote d'Ivoire’) OR TI,AB(‘Ivory Coast’) OR TI,AB(Gambia) OR TI,AB(Ghana) OR TI,AB(Guinea) OR TI,AB(Guinea-Bissau) OR TI,AB(Liberia) OR TI,AB(Mali) OR TI,AB(Mauritania) OR TI,AB(Niger) OR TI,AB(Nigeria) OR TI,AB(‘Sao Tome and Principe’) OR TI,AB(Senegal) OR TI,AB(‘Sierra Leone’) OR TI,AB(Togo) OR TI,AB(Burundi) OR TI,AB(Comoros) OR TI,AB(Djibouti) OR TI,AB(Eritrea) OR TI,AB(Ethiopia) OR TI,AB(Kenya) OR TI,AB(Madagascar) OR TI,AB(Malawi) OR TI,AB(Mozambique) OR TI,AB(Rwanda) OR TI,AB(Somalia) OR TI,AB(‘South Sudan’) OR TI,AB(Tanzania) OR TI,AB(Uganda) OR TI,AB(Zambia) OR TI,AB(Angola) OR TI,AB(‘Central African Republic’) OR TI,AB(Congo) OR TI,AB(‘DR Congo’) OR TI,AB(‘Congo Republic’) OR TI,AB(‘Democratic Republic of the Congo’) OR TI,AB(‘Equatorial Guinea’) OR TI,AB(Gabon))

Limits:

• Publication Type: Scholarly Journals, Working Papers

• Language: English

NHS EED

Date of Search: 10/3/2018

Search Terms:

(Afghanistan.af. OR Bahrain.af. OR Iran.af. OR Iraq.af. OR Jordan.af. OR Kuwait.af. OR Lebanon.af. OR Palestine.af. Oman.af. OR Qatar.af. OR Saudi Arabia.af. OR Turkey.af. OR United Arab Emirates.af. OR Yemen.af. OR Egypt.af. OR Libya.af. OR Morocco.af. OR Sudan.af. OR Syria.af. OR Tunisia.af. OR Botswana.af. OR Lesotho.af. OR Namibia.af. OR South Africa.af. OR Swaziland.af. OR Zimbabwe.af. OR Benin.af. OR Burkina Faso.af. OR Cameroon.af. OR Cape Verde.af. OR Chad.af. OR cote d'ivoire.af. OR The Gambia.af. OR Cabo Verde.af. OR Ivory Coast.af. OR Ghana.af. OR Guinea.af. OR Guinea-Bissau.af. OR Liberia.af. OR Mali.af. OR Mauritania.af. OR Niger.af. OR Nigeria.af. OR Sao Tome and Principe.af. OR Senegal.af. OR Sierra Leone.af. OR Togo.af. OR Burundi.af. OR Comoros.af. OR Djibouti.af. OR Eritrea.af. OR Ethiopia.af. OR Kenya.af. OR Madagascar.af. OR Malawi.af. OR Mozambique.af. OR Rwanda.af. OR Somalia.af. OR South Sudan.af. OR Tanzania.af. OR Uganda.af. OR Zambia.af. OR Angola.af. OR Central African Republic.af. OR Congo.af. OR Democratic Republic of the Congo.af. OR Equatorial Guinea.af. OR Gabon.af. OR Congo Republic.af. OR Africa.af.) AND (Quality-Adjusted Life Years/ OR DALY.mp. OR QALY.af. OR Disability-adjusted.af.)

Limits:

• None

**Appendix S3 – PRISMA diagram of included studies**

**Embase**

n = 8 studies

**Included in final sample**

**n = 358 studies**

**Included for full review**

n = 449 studies

**GH CEA Registry**

n = 268 studies

**CEA Registry**

n = 71 studies

**EconLit**

n = 1 studies

Note: The vast majority of studies identified (94%, n=339) were indexed in either the GH CEA Registry or CEA Registry. Only 6% of studies came from other sources (n=19).

Studies included in the final sample met the following criteria: (a) an original English-language article reporting at least one cost-effectiveness ratio; (b) published between 1975 and December 2017; (c) measured health outcomes in either QALY or DALY following the practice guidelines,3,31 and (d) focused on at least one African country or a sub-region (e.g., Sub-Saharan Africa).

**GH CEA registry**

n = 271 studies

**Other databases**

n = 839 studies

**CEA registry**

n = 141 studies

**NHS EED**

n = 7 studies

**Web of Science**

n = 3 studies

**All sources**

n = 1,251 studies

**n = 91 studies rejected**

- 67 had no African country
- 15 did not use cost-per-QALYs gained or DALYs averted
- 6 did not report a ratio
- 2 were abstracts
- 1 was a 2018 article

**n = 802 articles rejected**

- 421 duplicates
- 381 did not meet inclusion criteria (not original CEAs set in Africa reporting outcomes in QALYs or DALYs averted)

**Appendix S4– List of extracted variables**

*Author name*: The author name as reported in an article was extracted. Researchers then standardized names across articles so that the same author appearing in two different studies was identified by a single name in the dataset.

*Author affiliation*: an author’s institutional affiliation as reported in an article (i.e. Harvard University, World Health Organization, etc.). All “parent” institutional affiliations were extracted for each author. In other words, if an author reported multiple affiliations based at a single “parent” institution, the affiliation was only captured once (e.g., an author with two reported affiliations listed as “Harvard T.H. Chan School of Public Health” and “Harvard University” was assigned “Harvard University” as a single institutional affiliation). Researchers standardized affiliation names when possible so that the same institution was listed the same way across studies.

*Affiliation type*: the type of affiliation (government, academic, etc.)

*Affiliation country*: the country of the institutional affiliation as reported in an article. The same institution may have authors reporting different countries (i.e. one author affiliated with the WHO may be based out of Switzerland while another may be working in a country-based field office). We relied on the information available in each study to determine each author’s institution of affiliation and country of affiliation. We considered authors to have an “African–based affiliation” if the country of affiliation reported for their affiliated institution was a country in Africa. For example, since the US Centers for Disease Control and Prevention have programs and placements in Kenya and Uganda, we consider these to be “African-based affiliations.”

*Country of study*: the country the study examines. If a study evaluated more than one country, all countries were captured.

*Intervention type*: the type of intervention studied (pharmaceutical, immunization, etc.)

*Prevention level*: primary, secondary, or tertiary prevention

*Outcome measure*: the health outcome measure used in a particular study

*Disease area*: the disease area(s) studied (classified by 2016 GBD tier two categories)

*Funding source*: the type of funding supporting a study

*Probabilistic sensitivity analysis (PSA)*: did the authors perform a probabilistic sensitivity analysis

*Tufts CEA quality score*: a scale from 1 (low) to 7 (high) assessing the quality of articles indexed in the CEA and GH CEA registries. During data extraction, registry reviewers highly trained in cost-effectiveness independently assess the extent to which studies meet seven criteria while. Readers assign one point for each criterion met, with the final quality score assigned through a consensus process to resolve score discrepancies between readers. In some cases, a third reviewer may evaluate the study to help resolve discrepancies. All registry data including the quality score undergo a series of quality checks and cleaning to confirm the accuracy of the assessment. The quality criteria used are listed below:

1. Methods and results were communicated clearly and transparently to enable easy interpretation.
2. Time horizon was of sufficient length and discount rate appropriate.
3. Detailed disaggregated cost and QALY information was provided and recalculated ICER was correct (or did not differ by more than 10%).
4. Comprehensive characterization of uncertainty (sensitivity analyses).
5. Explicit reporting of utility weights (includes utility weight value and estimation method).
6. Subgroup analysis performed.
7. Non-health effects and/or spillover effects were quantified.

*iDSI reference case scores*: For a subset of cost-per-DALY studies, Tufts researchers developed an additional set of quality measures examining how adherent cost-per-DALY studies are to the iDSI Reference Case for Economic Evaluation (Emerson et. al. Adherence to the iDSI reference case among published cost-per-DALY averted studies. PloS one 2019; 14(5): e0205633.). The reference case provides standards for conducting economic evaluations across many different contexts, particularly low-and-middle-income countries such as those examined in the present work. The researchers translated these standards into a scoring system to assess how adherent studies were to reference case reporting standards and methodological standards. Studies receive one point for each standard met (there are 21 reporting standards and 19 methodological standards). Raw scores are converted to a percentage of total possible points, with 0% representing no adherence and 100% representing full adherence.

**Appendix S5 – Explanation of dyad generation**

Dyads represent pairs of two entities that share a common link. In our analysis, this link is the study. For each study in our sample, we generated dyads of collaboration at three different levels: 1) author, 2) affiliated institution, and 3) country of affiliated institution. In other words, we identified pairs of relationships for each of these levels for each study. We then determined the frequency with which each relationship occurred across all studies in our sample.

As a worked example, we selected three co-authors of our study to illustrate our approach to generate different dyads for network analysis.

a) Ari Panzer, Tufts Medical Center, United States

b) Avnee Patel, Health Intervention and Technology Assessment Program (HITAP), Ministry of Public Health, Nonthaburi, Thailand

c) Yot Teerawattananon, Health Intervention and Technology Assessment Program (HITAP), Ministry of Public Health, Nonthaburi, Thailand; The Saw Swee Hock School of Public Health, National University of Singapore, Singapore

Author dyads

Since these authors appear on the same study, a dyad is generated for each unique author relationship. Therefore, we end up with the following author dyads:

1) Panzer, Ari – Patel, Avnee

2) Panzer, Ari – Teerawattananon, Yot

3) Patel, Avnee – Teerawattananon, Yot

Institution dyads

Similarly, we generate a dyad at the institution level. If authors reported more than one affiliated institution, each would be used to generate dyads. We also included within-institution dyads. Therefore, we end up with the following institution dyads:

1) Tufts Medical Center – HITAP

2) Tufts Medical Center – National University of Singapore

3) HITAP – HITAP

4) HITAP - National University of Singapore

Country dyads

Finally, a country-level dyad is generated for each country of affiliated institution.

1) United States – Thailand

2) United States – Singapore

3) Thailand – Singapore

Using the unique measure of the network at the author, institution, and county levels, we could examine patterns of collaboration and understand the extent of individual authors, between institutions, cross-national, and within-Africa collaboration.

**Appendix S6: Supplementary figures**

**Figure A – Collaboration patterns among contributing authors based on global regions**

**
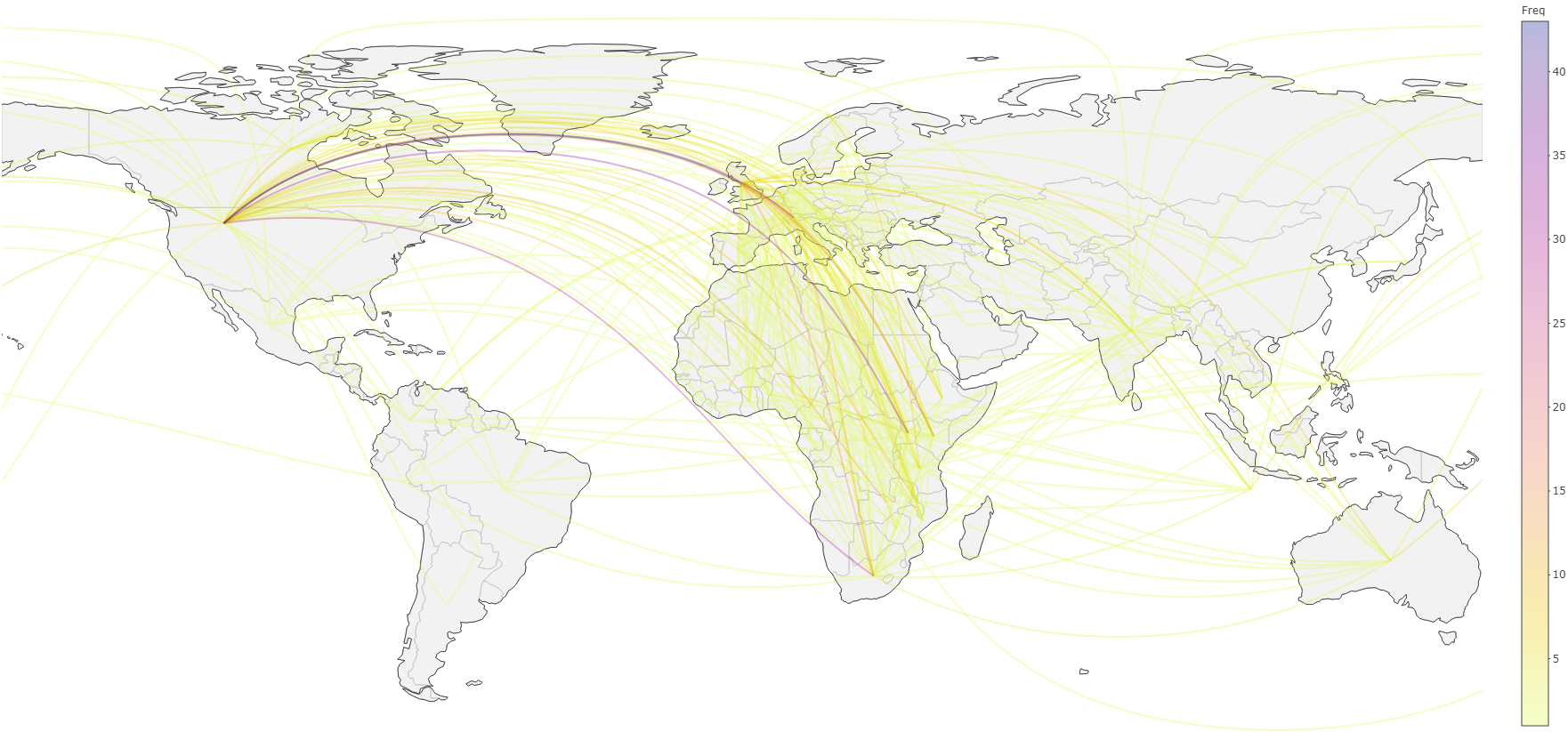
**Note: Lines connect based on mutual authorship on papers (i.e. if co-authors from the United States and Uganda work together, a line would connect the US to Uganda). Connection points on countries represent the geographical center of each country (i.e. for the US, the geographic center is pulled north and west by the inclusion of Alaska). The color of the lines represents the relative frequency of a particular collaboration (see color bar on right hand side). Dark purple and red represent high-frequency collaborations, orange represents medium-frequency collaborations, and yellow represents low-frequency collaborations.

**Figure B – Research-level collaboration between African countries**

**
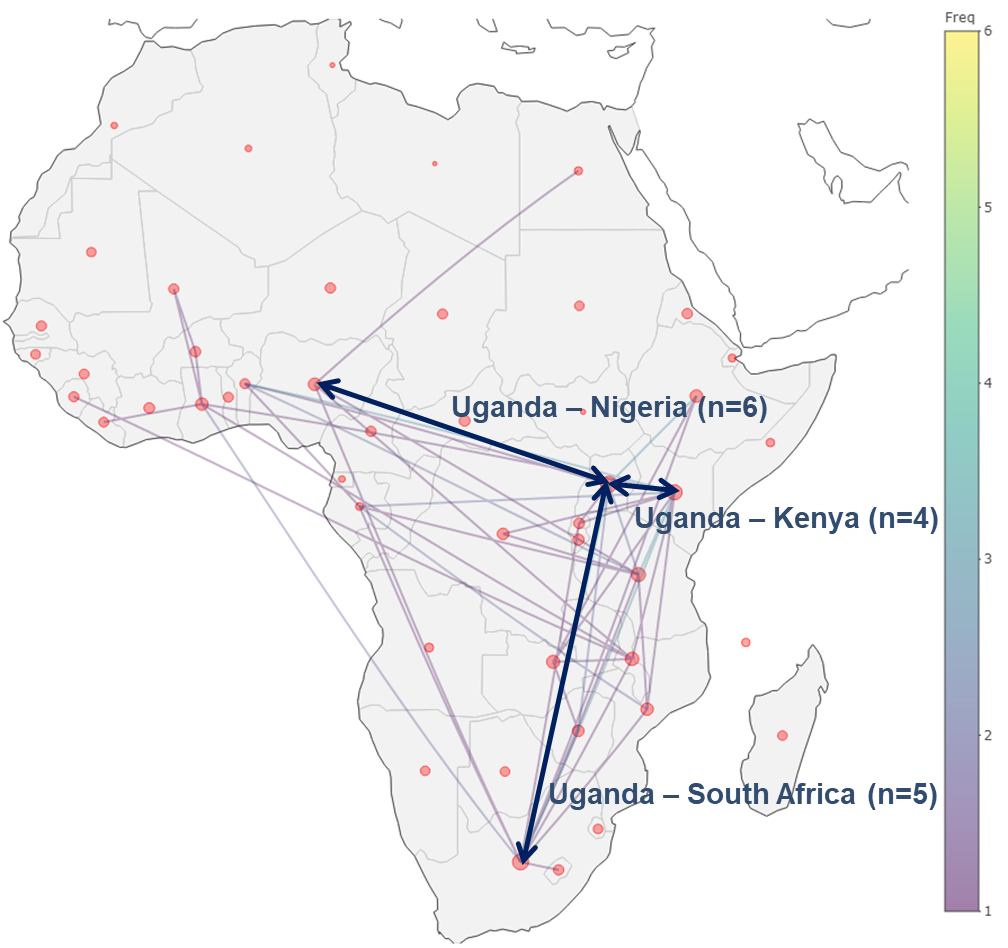
**

Note: The figure describes the researcher-level collaboration between African countries. We define the researcher-level collaboration based on author’s affiliated institution and the location of the institution. Red dots are proportional to the number of studies relevant to a specific country and placed on the geographical center of each country. The color of the lines represents the relative frequency of a particular collaboration (see color bar on right hand side). To better visualize, we highlighted the top three collaboration between African countries in this Figure.

**Figure C** – **Number of cost-per-DALY studies by country in Africa**


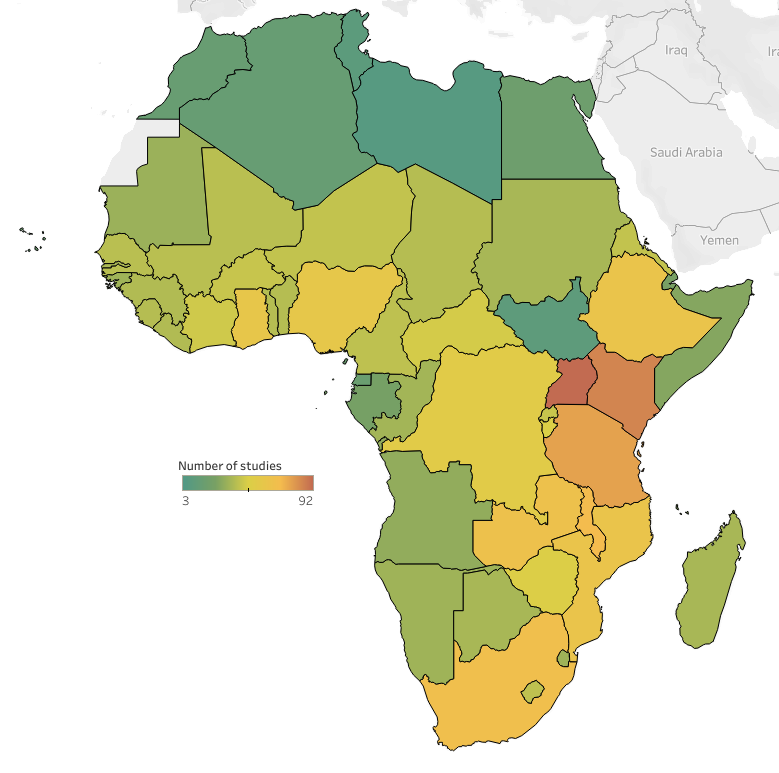


Note: In this heat map, green indicates a relatively low number of studies while red indicates a relatively high number of studies. Cost-per-DALY studies tend to be concentrated in Sub-Saharan Africa. Uganda (n=92) and Kenya (n=85) had the most studies (See Online Appendix S7: Table A for full results).

**Figure D** – **Number of cost-per-QALY studies by country in Africa**


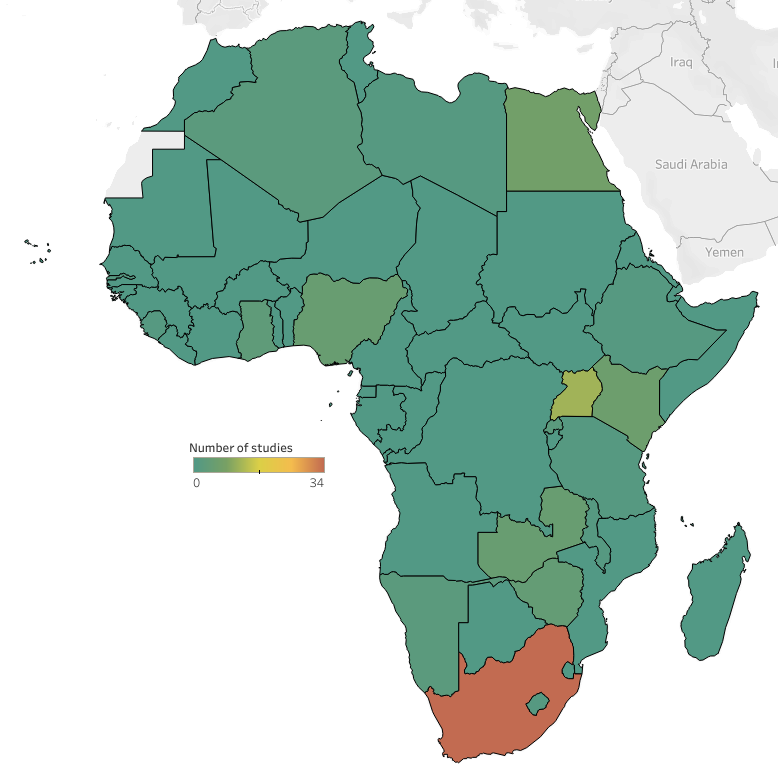


Note: In this heat map, green indicates a relatively low number of studies while red indicates a relatively high number of studies. Cost-per-QALY studies are most often available for South Africa (n=32 and Uganda (n=12). Most countries have no studies available (See Online Appendix S7: Table A for full results).

**Appendix S7: Supplementary Tables**

| **Table A. African Countries Ranked by Number of Studies** | | | | | | |
| --- | --- | --- | --- | --- | --- | --- |
|  | Cost/DALY (n=274) | | Cost/QALY (n=88) | | Total (n=358) | |
| Country* | Number of studies | % | Number of studies | % | Number of studies | % |
| Uganda | 92 | 33% | 12 | 14% | 103 | 29% |
| South Africa | 67 | 24% | 34 | 39% | 101 | 28% |
| Kenya | 85 | 31% | 6 | 7% | 91 | 25% |
| Tanzania | 77 | 28% | 1 | 1% | 78 | 22% |
| Malawi | 70 | 25% | 1 | 1% | 71 | 20% |
| Zambia | 64 | 23% | 5 | 6% | 68 | 19% |
| Nigeria | 58 | 21% | 5 | 6% | 63 | 18% |
| Ethiopia | 61 | 22% | 1 | 1% | 62 | 17% |
| Ghana | 59 | 21% | 3 | 3% | 61 | 17% |
| Mozambique | 61 | 22% | 0 | 0% | 61 | 17% |
| Democratic Republic of the Congo | 53 | 19% | 0 | 0% | 53 | 15% |
| Zimbabwe | 49 | 18% | 4 | 5% | 53 | 15% |
| Burundi | 48 | 17% | 0 | 0% | 48 | 13% |
| Central African Republic | 46 | 17% | 0 | 0% | 46 | 13% |
| Côte d'Ivoire | 45 | 16% | 0 | 0% | 45 | 13% |
| Rwanda | 43 | 16% | 2 | 2% | 45 | 13% |
| Burkina Faso | 43 | 16% | 0 | 0% | 43 | 12% |
| Eritrea | 42 | 15% | 0 | 0% | 42 | 12% |
| Lesotho | 41 | 15% | 1 | 1% | 42 | 12% |
| Niger | 42 | 15% | 0 | 0% | 42 | 12% |
| Cameroon | 41 | 15% | 0 | 0% | 41 | 11% |
| Mali | 40 | 15% | 0 | 0% | 40 | 11% |
| Senegal | 40 | 15% | 0 | 0% | 40 | 11% |
| Sierra Leone | 39 | 14% | 1 | 1% | 40 | 11% |
| Benin | 39 | 14% | 0 | 0% | 39 | 11% |
| Chad | 39 | 14% | 0 | 0% | 39 | 11% |
| Togo | 39 | 14% | 0 | 0% | 39 | 11% |
| Guinea | 38 | 14% | 0 | 0% | 38 | 11% |
| Liberia | 38 | 14% | 0 | 0% | 38 | 11% |
| Gambia | 37 | 13% | 1 | 1% | 37 | 10% |
| Botswana | 36 | 13% | 0 | 0% | 36 | 10% |
| Madagascar | 36 | 13% | 0 | 0% | 36 | 10% |
| Namibia | 34 | 12% | 2 | 2% | 36 | 10% |
| Sudan | 36 | 13% | 0 | 0% | 36 | 10% |
| Congo Rep | 35 | 13% | 0 | 0% | 35 | 10% |
| Guinea-Bissau | 34 | 12% | 0 | 0% | 34 | 9% |
| Swaziland | 34 | 12% | 0 | 0% | 34 | 9% |
| Mauritania | 33 | 12% | 0 | 0% | 33 | 9% |
| Angola | 31 | 11% | 0 | 0% | 31 | 9% |
| Comoros | 28 | 10% | 0 | 0% | 28 | 8% |
| Somalia | 28 | 10% | 0 | 0% | 28 | 8% |
| Egypt | 19 | 7% | 7 | 8% | 26 | 7% |
| Djibouti | 25 | 9% | 0 | 0% | 25 | 7% |
| Gabon | 24 | 9% | 0 | 0% | 24 | 7% |
| Sao Tome and Principe | 23 | 8% | 0 | 0% | 23 | 6% |
| Cabo Verde | 18 | 7% | 0 | 0% | 18 | 5% |
| Algeria | 15 | 5% | 2 | 2% | 17 | 5% |
| Equatorial Guinea | 17 | 6% | 0 | 0% | 17 | 5% |
| Morocco | 16 | 6% | 0 | 0% | 16 | 4% |
| South Sudan | 10 | 4% | 0 | 0% | 10 | 3% |
| Could not be determined^^^ | 3 | 1% | 6 | 7% | 9 | 3% |
| Tunisia | 9 | 3% | 0 | 0% | 9 | 3% |
| Libya | 6 | 2% | 1 | 1% | 7 | 2% |

*****Not mutually exclusive (i.e., studies could evaluate more than one country)

^^^Studies categorized as “could not be determined” studied an African sub-region (e.g., Western Sub-Saharan Africa) but did not report the specific countries analyzed.

| **Table B. Intervention-Specific Cost-Effectiveness Ratios** | | | | | |
| --- | --- | --- | --- | --- | --- |
| **Cost-per-DALY averted** | | | | | |
|  | Number of ratios  (% of total) | Number of Cost-saving  (% of total) | Median ICERs^#^ | IQR | Number of Dominated  (% of total) |
| **All ratios** | 1891  (100%) | 129 of 1891  (7%) | 170 | (46, 815) | 14 of 1891  (1%) |
| **Intervention*** |  |  |  |  |  |
| Maternal/Neonatal | 221 (12%) | 7 | 54 | (15, 317) | 3 |
| Others | 710 (38%) | 23 | 115 | (36, 503) | 1 |
| Immunization | 568 (30%) | 62 | 141 | (54, 620) | 1 |
| Health Education or Behavior | 355 (19%) | 6 | 176 | (67, 764) | 0 |
| Pharmaceutical | 819 (43%) | 34 | 272.5 | (50, 1418) | 11 |
| Screening | 371 (20%) | 48 | 295 | (84.5, 1206) | 3 |
| Surgical | 236 (13%) | 1 | 329.5 | (76, 1120) | 0 |
| Care Delivery | 255 (14%) | 9 | 333 | (34, 3247) | 0 |
| Diagnostic | 137 (7%) | 12 | 352 | (60.5, 1303) | 1 |
| **Cost-per-QALY** | | | | | |
|  | Number of ratios  (% of total) | Number of Cost-saving  (% of total) | Median ICERs^#^ | IQR | Number of Dominated  (% of total) |
| **All ratios** | 251  (100%) | 34 of 251  (14%) | 1532 | (238, 6451) | 26 of 251  (10%) |
| **Intervention*** |  |  |  |  |  |
| Surgical | 12 (5%) | 2 | 181.5 | (36, 5121) | 0 |
| Maternal/Neonatal | 1 (<1%) | 0 | 249 | N/A | 0 |
| Diagnostic | 23 (10%) | 6 | 272 | (235, 10058) | 2 |
| Immunization | 8 (3%) | 0 | 320 | (38, 1726) | 1 |
| Screening | 48 (20%) | 2 | 1237 | (243, 2821) | 0 |
| Pharmaceutical | 139 (55%) | 21 | 1470 | (216, 6196) | 21 |
| Others | 68 (28%) | 10 | 1547.5 | (575, 6451) | 4 |
| Health Education or Behavior | 17 (7%) | 1 | 2204.5 | (926, 5052) | 0 |
| Care Delivery | 20 (8%) | 3 | 2642 | (2039, 5272) | 0 |

Note: Among 2,142 intervention-specific cost-effectiveness ratios extracted from 358 studies, 8% (n=163) were deemed cost-saving, and most of these evaluated immunizations, pharmaceuticals, or screening strategies. A greater proportion of cost-per-QALY ratios were reported to be cost-saving compared to cost-per-DALY ratios (n=34, 14% vs. n=129, 7%).
*Intervention types are not mutually exclusive categories
^#^Cost-saving and dominated interventions are excluding from the median incremental cost-effectiveness ratio (ICER) calculations

| **Table C. Top 25 Country Dyads by Frequency of Collaboration*** | |
| --- | --- |
| **Country Dyad** | **Frequency of Collaboration** |
| United States – United States | 144 |
| United Kingdom – United Kingdom | 55 |
| United States – United Kingdom | 43 |
| South Africa – South Africa | 35 |
| United States – Uganda | 33 |
| United States – South Africa | 29 |
| Switzerland – Switzerland | 29 |
| United States – Switzerland | 28 |
| Uganda – Uganda | 24 |
| Kenya – Kenya | 23 |
| United Kingdom – Switzerland | 19 |
| Netherlands – Netherlands | 17 |
| United States – Kenya | 16 |
| Nigeria – Nigeria | 15 |
| United Kingdom – South Africa | 15 |
| United Kingdom – Netherlands | 13 |
| Tanzania – Tanzania | 11 |
| Zambia – United States | 11 |
| United States – Canada | 10 |
| Egypt – Egypt | 9 |
| United States – Ghana | 9 |
| United Kingdom – Kenya | 9 |
| United States – Netherlands | 9 |
| Switzerland – South Africa | 9 |
| Zambia – Zambia | 9 |

*****See Appendix S5 for an explanation and example of dyad generation

| **Table D. Top 25 Institution Dyads by Frequency of Collaboration** | |
| --- | --- |
| **Institution Dyad** | **Frequency of Collaboration** |
| London School of Hygiene & Tropical Medicine – London School of Hygiene & Tropical Medicine | 21 |
| Harvard University – Harvard University | 18 |
| Johns Hopkins University – Johns Hopkins University | 18 |
| World Health Organization – World Health Organization | 16 |
| Makerere University – Makerere University | 15 |
| University of Washington – University of Washington | 15 |
| Centers for Disease Control and Prevention – Centers for Disease Control and Prevention | 14 |
| University of the Witwatersrand – University of the Witwatersrand | 13 |
| University of Cape Town – University of Cape Town | 12 |
| Imperial College London – Imperial College London | 9 |
| Swiss Tropical and Public Health Institute – Swiss Tropical and Public Health Institute | 9 |
| Makerere University – Johns Hopkins University | 8 |
| World Health Organization – London School of Hygiene & Tropical Medicine | 8 |
| Program for Appropriate Technologies in Health (PATH) – Program for Appropriate Technologies in Health (PATH) | 8 |
| University of Basel – Swiss Tropical and Public Health Institute | 7 |
| University of Bergen – University of Bergen | 7 |
| London School of Hygiene & Tropical Medicine – Centers for Disease Control and Prevention | 6 |
| London School of Hygiene & Tropical Medicine – Imperial College London | 6 |
| University of the Witwatersrand – London School of Hygiene & Tropical Medicine | 6 |
| New York University – New York University | 6 |
| University College London – University College London | 6 |
| Makerere University – Bayero University | 5 |
| Harvard University – Boston Children's Hospital | 5 |
| Harvard University – Brigham and Women's Hospital | 5 |
| Seven other dyad pairs tied | 5 |

*****See Appendix S5 for an explanation and example of dyad generation

| **Table E. Top 25 Author Dyads by Frequency of Collaboration*** | |
| --- | --- |
| **Author Dyad** | **Frequency of Collaboration** |
| Lamorde, Mohammed – Kuznik, Andreas | 7 |
| Kuznik, Andreas – Habib, Abdulrazaq G | 5 |
| Lamorde, Mohammed – Habib, Abdulrazaq G | 5 |
| Celum, Connie L – Baeten, Jared M | 4 |
| Marseille, Elliot – Kahn, James G | 4 |
| Alonso, Pedro L – Menéndez, Clara | 3 |
| Baeten, Jared M – Barnabas, Ruanne V | 3 |
| Braithwaite, Ronald Scott – Nucifora, Kimberly A | 3 |
| Celum, Connie L – Barnabas, Ruanne V | 3 |
| Gargano, Lisa M – Cookson, Susan T | 3 |
| Garrison Jr., Louis P – Babigumira, Joseph B | 3 |
| Manabe, Yukari C – Castelnuovo, Barbara | 3 |
| Ngalesoni, Frida N – Robberstad, Bjarne | 3 |
| Nucifora, Kimberly A – Kessler, Jason | 3 |
| O'Shea, Meredith – Diaz, Mireia | 3 |
| Rheingans, Richard D – Atherly, Deborah E | 3 |
| Robberstad, Bjarne – Norheim, Ole Frithjof | 3 |
| Ruhago, George M – Ngalesoni, Frida N | 3 |
| Shah, Maunank – Manabe, Yukari C | 3 |
| Sharma, Monisha – Baeten, Jared M | 3 |
| Sharma, Monisha – Barnabas, Ruanne V | 3 |
| Sharma, Monisha – Celum, Connie L | 3 |
| White, Richard G – Vassall, Anna | 3 |
| Ying, Roger – Barnabas, Ruanne V | 3 |
| Ying, Roger – Sharma, Monisha | 3 |

*****See Appendix S5 for an explanation and example of dyad generation

| **Table F. African Institutions Ranked by Number of Studies** | | |
| --- | --- | --- |
| African Institution | Number of studies | % of total studies (n=358) |
| Makerere University | 25 | 7% |
| University of Cape Town | 23 | 6% |
| University of the Witwatersrand | 20 | 6% |
| Kenya Medical Research Institute | 7 | 2% |
| Tanzania Ministry of Health, Community Development, Gender, Elders and Children | 7 | 2% |
| Centers for Disease Control and Prevention | 6 | 2% |
| Kenya Ministry of Health | 6 | 2% |
| University of Zimbabwe | 6 | 2% |
| World Health Organization | 6 | 2% |
| Amref Health Africa | 5 | 1% |
| Bayero University | 5 | 1% |
| Ifakara Health Institute | 5 | 1% |
| Independent Consultant | 5 | 1% |
| Muhimbili University of Health and Allied Sciences | 5 | 1% |
| National Institute for Medical Research | 5 | 1% |
| University of Ghana | 5 | 1% |
| University of Malawi | 5 | 1% |
| Zambia Ministry of Health | 5 | 1% |
| Centre for Infectious Disease Research in Zambia | 4 | 1% |
| Kenya Medical Research Institute-Wellcome Trust Collaborative Research Programme | 4 | 1% |
| Malawi Ministry of Health | 4 | 1% |
| Manhiça Health Research Center | 4 | 1% |
| Mbarara University of Science and Technology | 4 | 1% |
| South African Medical Research Council | 4 | 1% |
| University of Nairobi | 4 | 1% |
| Ain Shams University | 3 | 1% |
| Egyptian Ministry of Health and Population | 3 | 1% |
| Ghana Health Service | 3 | 1% |
| Groote Schuur Hospital | 3 | 1% |
| HEXOR (Pty) Ltd. | 3 | 1% |
| Human Sciences Research Council | 3 | 1% |
| Joint Clinical Research Centre | 3 | 1% |
| Kenya Medical Research Institute and Centers for Disease Control and Prevention Research and Public Health Collaboration | 3 | 1% |
| Kwame Nkrumah University of Science and Technology | 3 | 1% |
| Medical Research Council Unit The Gambia at the London School of Hygiene & Tropical Medicine | 3 | 1% |
| Mozambique Ministry of Health | 3 | 1% |
| Nnamdi Azikiwe University | 3 | 1% |
| South African National Department of Health | 3 | 1% |
| The British University in Egypt | 3 | 1% |
| Uganda Ministry of Health | 3 | 1% |
| United States Agency for International Development | 3 | 1% |
| University College Hospital, Ibadan | 3 | 1% |
| University of KwaZulu-Natal | 3 | 1% |
| University of Nigeria, Nsukka | 3 | 1% |
| Addis Ababa University | 2 | 1% |
| Aurum Institute | 2 | 1% |
| Benin Ministry of Health | 2 | 1% |
| Bugando Medical Centre | 2 | 1% |
| Center for Evidence-Based Global Health | 2 | 1% |
| Central Administration for Pharmaceutical Affairs | 2 | 1% |
| Centro de Investigação em Saúde de Manhiça | 2 | 1% |
| Fayoum University | 2 | 1% |
| Haydom Lutheran Hospital | 2 | 1% |
| Hygeia Nigeria Ltd | 2 | 1% |
| INDEPTH Network | 2 | 1% |
| International Livestock Research Institute | 2 | 1% |
| International Water Management Institute | 2 | 1% |
| Kenyatta National Hospital | 2 | 1% |
| Management Sciences for Health | 2 | 1% |
| Médecins Sans Frontiéres | 2 | 1% |
| Moi University | 2 | 1% |
| Mulago National Referral Hospital | 2 | 1% |
| National Liver Institute, Menoufiya University | 2 | 1% |
| National Malaria Elimination Centre | 2 | 1% |
| Navrongo Health Research Centre | 2 | 1% |
| Ogo Oluwa Hospital | 2 | 1% |
| Program for Appropriate Technologies in Health (PATH) | 2 | 1% |
| South African Centre for Epidemiological Modelling and Analysis | 2 | 1% |
| Tanzania National Institute for Medical Research | 2 | 1% |
| Tumaini University Makumira | 2 | 1% |
| Uganda Virus Research Institute | 2 | 1% |
| University of Health and Allied Sciences | 2 | 1% |
| University of Ilorin | 2 | 1% |
| University of Ilorin Teaching Hospital | 2 | 1% |
| University of Stellenbosch | 2 | 1% |
| University of Zambia | 2 | 1% |
| Zambia Centre for Applied Health Research and Development | 2 | 1% |
| Abu Homos District Hospital | 1 | 0% |
| Academic Model Providing Access to Healthcare | 1 | 0% |
| Addis Ababa City Government Health Bureau | 1 | 0% |
| Africa Centre | 1 | 0% |
| African Programme for Onchocerciasis Control | 1 | 0% |
| AIDS Information Centre | 1 | 0% |
| Albert Royer Children's Hospital | 1 | 0% |
| Aminu Kano Teaching Hospital | 1 | 0% |
| Assiut University Women's Health Center | 1 | 0% |
| Axios International | 1 | 0% |
| BasicNeeds | 1 | 0% |
| Beit CURE Hospital | 1 | 0% |
| Biomedical Research and Training Institute and University of Zimbabwe College of Health Sciences | 1 | 0% |
| Black Lion Hospital | 1 | 0% |
| Bo District Hospital | 1 | 0% |
| Boehringer Ingelheim | 1 | 0% |
| Bowen University Teaching Hospital | 1 | 0% |
| Brenthurst Clinic | 1 | 0% |
| Butabika National Referral Hospital for Mental, Neurological and Substance Abuse Disorders | 1 | 0% |
| Cairo University | 1 | 0% |
| Canadian International College | 1 | 0% |
| Center for the AIDS Program of Research in South Aftrica | 1 | 0% |
| Centre de Support en Santé Internationale | 1 | 0% |
| Centre Suisse de Recherches Scientifi ques en Côte d'Ivoire | 1 | 0% |
| Chronic Diseases Initiative for Africa | 1 | 0% |
| Community of Sant'Egidio | 1 | 0% |
| Connaught Hospital | 1 | 0% |
| Could not be determined | 1 | 0% |
| Desmond Tutu HIV Foundation | 1 | 0% |
| Dodowa Health Research Centre | 1 | 0% |
| DREAM programme-Community of Sant'Egidio | 1 | 0% |
| Edendale Hospital | 1 | 0% |
| Egerton University | 1 | 0% |
| El Galaa Maternity Teaching Hospital | 1 | 0% |
| EngenderHealth | 1 | 0% |
| Epicentre Uganda | 1 | 0% |
| Ethiopia Federal Ministry of Health | 1 | 0% |
| Fatick Hospital | 1 | 0% |
| Federal Teaching Hospital, Gombe | 1 | 0% |
| FHI 360 | 1 | 0% |
| Gabriel Touré Teaching Hospital | 1 | 0% |
| German University in Cairo | 1 | 0% |
| Gharbiya Health Directorate | 1 | 0% |
| Global Communities | 1 | 0% |
| Goderich Emergency Hospital | 1 | 0% |
| HIV Vaccine Trials Network | 1 | 0% |
| Hôpital de District de Kolofata | 1 | 0% |
| Hôpital Principal de Dakar | 1 | 0% |
| Iganga/Mayuge Health and Demographic Surveillance Site | 1 | 0% |
| International Center for Tropical Agriculture | 1 | 0% |
| International Institute of Tropical Agriculture | 1 | 0% |
| International Maize and Wheat Improvement Center | 1 | 0% |
| Japan International Cooperation Agency | 1 | 0% |
| Jhpiego | 1 | 0% |
| Joint Malaria Program | 1 | 0% |
| Jos University Teaching Hospital | 1 | 0% |
| Kadic Hospital | 1 | 0% |
| Karaye Hospital | 1 | 0% |
| Katsina General Hospital | 1 | 0% |
| Kenya Association of Professional Counselors | 1 | 0% |
| Kenya Bureau of Standards | 1 | 0% |
| Kijabe Hospital | 1 | 0% |
| Kilembe Mines Hospital | 1 | 0% |
| Kilimanjaro Christian Medical Centre | 1 | 0% |
| Kilimanjaro Women Against AIDS | 1 | 0% |
| Kisumu Medical and Educational Trust | 1 | 0% |
| Laboratoire de Recherches Vétérinaire et Zootechnique | 1 | 0% |
| Le Centre pour le Développement des Vaccins du Mali | 1 | 0% |
| LifeNet International | 1 | 0% |
| Lighthouse Trust | 1 | 0% |
| London School of Hygiene & Tropical Medicine | 1 | 0% |
| Lufwanyama District Health Management Team | 1 | 0% |
| Macha Mission Hospital and Macha Research Trust | 1 | 0% |
| Malawi College of Health Sciences | 1 | 0% |
| Malawi Epidemiology and Intervention Research Unit | 1 | 0% |
| Malawi-Liverpool-Wellcome Trust Clinical Research Programme | 1 | 0% |
| Maputo Central Hospital | 1 | 0% |
| Masinde Muliro University of Science and Technology | 1 | 0% |
| Mbarara Regional Referral Hospital | 1 | 0% |
| MEASURE Evaluation | 1 | 0% |
| Menofiya University | 1 | 0% |
| MyungSung Christian Medical Center | 1 | 0% |
| National Agency for the Control of AIDS | 1 | 0% |
| National Blood Service Zimbabwe | 1 | 0% |
| National Blood Transfusion Service | 1 | 0% |
| National Directorate of Health | 1 | 0% |
| National Food and Nutrition Commission | 1 | 0% |
| National Hepatology and Tropical Medicine Research Institute | 1 | 0% |
| National Institute for Communicable Diseases | 1 | 0% |
| National Orthopaedic Hospital | 1 | 0% |
| National Primary Health Care Development Agency | 1 | 0% |
| National Tuberculosis and Leprosy Programme | 1 | 0% |
| New HIV Vaccine and Microbicide Advocacy Society | 1 | 0% |
| North-West University | 1 | 0% |
| Nutrition International | 1 | 0% |
| Nyanza Provincial General Hospital | 1 | 0% |
| Obafemi Awolowo University | 1 | 0% |
| Office of President Cabinet | 1 | 0% |
| Parent and Child Health Initiative | 1 | 0% |
| Pemba Operational Research Nucleus | 1 | 0% |
| Rwanda Military Hospital | 1 | 0% |
| Sabin Vaccine Institute | 1 | 0% |
| Saint Francis Designated District Hospital | 1 | 0% |
| Scott Hospital | 1 | 0% |
| Senegal Ministry of Health | 1 | 0% |
| SolidarMed | 1 | 0% |
| South African National AIDS Council | 1 | 0% |
| Specialist Hospital Sokoto | 1 | 0% |
| St Francis' Hospital | 1 | 0% |
| Stellenbosch University | 1 | 0% |
| Tanta University | 1 | 0% |
| The AIDS Support Organization | 1 | 0% |
| The National Food and Nutrition Commission | 1 | 0% |
| The Salvation Army | 1 | 0% |
| The Save the Children Fund | 1 | 0% |
| The Technical University of Kenya | 1 | 0% |
| The United Nations International Children's Emergency Fund | 1 | 0% |
| Thyolo District Hospital | 1 | 0% |
| Togolese Ministry of Health | 1 | 0% |
| Tropical Diseases Research Centre | 1 | 0% |
| Uganda Martyrs University | 1 | 0% |
| United States Department of Defense | 1 | 0% |
| Université d'Abomey-Calavi | 1 | 0% |
| Université Félix Houphouët-Boigny | 1 | 0% |
| University for Development Studies | 1 | 0% |
| University Hospital of Sétif | 1 | 0% |
| University of Abuja | 1 | 0% |
| University of Addis Abeba | 1 | 0% |
| University of Bamako | 1 | 0% |
| University of Dar es Salaam | 1 | 0% |
| University of Ibadan | 1 | 0% |
| University of Jos | 1 | 0% |
| University of Kinshasa | 1 | 0% |
| University of Ouagadougou | 1 | 0% |
| University of Pretoria | 1 | 0% |
| University of Rwanda | 1 | 0% |
| University of Sierra Leone | 1 | 0% |
| University of the Free State | 1 | 0% |
| University Research Co. | 1 | 0% |
| University Teaching Hospital | 1 | 0% |
| University Teaching Hospital and University of Zambia | 1 | 0% |
| US Naval Medical Research Unit No. 3 | 1 | 0% |
| Usmanu Danfodiyo University | 1 | 0% |
| World Vision International | 1 | 0% |
| Zanzibar Ministry of Health | 1 | 0% |
| Zimbabwe Ministry of Health and Child Care | 1 | 0% |

**Table G. Top African Institutions vs Others**

|  | **Top Institution Studies (n=55)^#^** | | **Other Institution Studies (n=156)^#^** | | **Top vs. Other Institutions** | **All Studies with a Quality Score (n=211)^#^** | |
| --- | --- | --- | --- | --- | --- | --- | --- |
|  | Number of studies | % of the sample | Number of studies | % of the sample | p-value | Number of studies | % of the sample |
| **All studies** | 55 | 26% | 156 | 74% |  | 211 | 100% |
| **Health Outcome Measure^** |  |  |  |  |  |  |  |
| Cost/DALY | 37 | 67% | 134 | 86% | <.01 | 171 | 81% |
| Cost/QALY | 19 | 35% | 24 | 15% | <.01 | 43 | 20% |
| **GBD Super Region^** |  |  |  |  |  |  |  |
| Sub-Saharan Africa | 55 | 100% | 150 | 96% | 0.14 | 205 | 97% |
| North Africa | 3 | 5% | 11 | 7% | 0.68 | 14 | 7% |
| **Prevention Level^** |  |  |  |  |  |  |  |
| Primary | 17 | 31% | 79 | 51% | <.05 | 96 | 45% |
| Secondary | 15 | 27% | 24 | 15% | 0.051 | 39 | 18% |
| Tertiary | 28 | 51% | 71 | 46% | 0.49 | 99 | 47% |
| **Top GBD Disease Categories^** |  |  |  |  |  |  |  |
| HIV/AIDS and tuberculosis | 29 | 53% | 49 | 31% | <.01 | 78 | 37% |
| Other communicable, maternal, neonatal, and nutritional disorders | 5 | 9% | 16 | 10% | 0.804 | 21 | 10% |
| Cardiovascular and circulatory diseases | 4 | 7% | 8 | 5% | 0.555 | 12 | 6% |
| Diarrhea, lower respiratory infections, meningitis, and other common infectious diseases | 3 | 5% | 22 | 14% | 0.088 | 25 | 12% |
| Maternal disorders | 3 | 5% | 9 | 6% | 0.931 | 12 | 6% |
| Neglected tropical diseases and malaria | 2 | 4% | 26 | 17% | <.05 | 28 | 13% |
| Neoplasms | 2 | 4% | 2 | 1% | 0.271 | 4 | 2% |
| Diabetes, urogenital, blood, and endocrine diseases | 2 | 4% | 2 | 1% | 0.271 | 4 | 2% |
| Nutritional deficiencies | 0 | 0% | 5 | 3% | 0.179 | 5 | 2% |
| Other non-communicable diseases | 0 | 0% | 5 | 3% | 0.179 | 5 | 2% |
| Other diseases | 8 | 15% | 23 | 15% | 0.972 | 31 | 15% |
| **Top Interventions^** |  |  |  |  |  |  |  |
| Pharmaceutical | 31 | 56% | 69 | 44% | 0.121 | 100 | 47% |
| Care Delivery | 10 | 18% | 30 | 19% | 0.864 | 40 | 19% |
| Screening | 9 | 16% | 20 | 13% | 0.512 | 29 | 14% |
| Maternal and Neonatal | 6 | 11% | 20 | 13% | 0.742 | 26 | 12% |
| Immunization | 5 | 9% | 29 | 19% | 0.099 | 34 | 16% |
| Health Education or Behavior | 5 | 9% | 22 | 14% | 0.339 | 27 | 13% |
| Diagnostic | 5 | 9% | 10 | 6% | 0.506 | 15 | 7% |
| Surgical | 2 | 4% | 12 | 8% | 0.299 | 14 | 7% |
| Other Interventions | 6 | 11% | 41 | 26% | <.05 | 47 | 22% |
| **Study Sponsor^** |  |  |  |  |  |  |  |
| Government/Academic | 26 | 47% | 88 | 56% | 0.242 | 114 | 54% |
| Foundation | 21 | 38% | 53 | 34% | 0.574 | 74 | 35% |
| Could not be determined/None | 15 | 27% | 24 | 15% | 0.051 | 39 | 18% |
| Pharma/Device Company | 7 | 13% | 10 | 6% | 0.139 | 17 | 8% |
| Other | 5 | 9% | 28 | 18% | 0.12 | 33 | 16% |
| **Probabilistic Sensitivity Analysis** | 33 | 60% | 65 | 42% | <.05 | 98 | 46% |

^^^Because a quality score for CEAs is only available for the study sample in the CEA or Global Health CEA registries (59% of the total sample and 92% of the sample with an African institution), this analysis includes only studies that included a quality score from the CEA or GH CEA registries. Studies co-authored by the top three African institutions (those contributing to more than 10 studies) are considered “Top African Institution Studies” while all other studies co-authored by other African institutions are considered “Other African Institution Studies.”

*Not mutually exclusive categories (i.e., one study could be counted in more than one category)

^#^Proportions are based on the total number of studies in each sample
